# Extracellular Vesicles in Amyotrophic Lateral Sclerosis

**DOI:** 10.3390/life13010121

**Published:** 2022-12-31

**Authors:** Gavin McCluskey, Karen E. Morrison, Colette Donaghy, Frederique Rene, William Duddy, Stephanie Duguez

**Affiliations:** 1 Personalised Medicine Centre, School of Medicine, Ulster University, Derry BT47 6SB, UK; 2 Department of Neurology, Altnagelvin Hospital, Derry BT47 6SB, UK; 3 Department of Neurology, Royal Victoria Hospital, Belfast BT12 6BA, UK; 4 Faculty of Medicine, Health & Life Sciences, Queen’s University, Belfast BT9 6AG, UK; 5 INSERM U1118, Centre de Recherche en Biomédecine de Strasbourg, Université de Strasbourg, 67000 Strasbourg, France

**Keywords:** exosomes, extracellular vesicles, amyotrophic lateral sclerosis, motor neuron disease

## Abstract

Amyotrophic Lateral Sclerosis is a progressive neurodegenerative disease and is the most common adult motor neuron disease. The disease pathogenesis is complex with the perturbation of multiple pathways proposed, including mitochondrial dysfunction, RNA processing, glutamate excitotoxicity, endoplasmic reticulum stress, protein homeostasis and endosomal transport/extracellular vesicle (EV) secretion. EVs are nanoscopic membrane-bound particles that are released from cells, involved in the intercellular communication of proteins, lipids and genetic material, and there is increasing evidence of their role in ALS. After discussing the biogenesis of EVs, we review their roles in the propagation of pathological proteins in ALS, such as TDP-43, SOD1 and FUS, and their contribution to disease pathology. We also discuss the ALS related genes which are involved in EV formation and vesicular trafficking, before considering the EV protein and RNA dysregulation found in ALS and how these have been investigated as potential biomarkers. Finally, we highlight the potential use of EVs as therapeutic agents in ALS, in particular EVs derived from mesenchymal stem cells and EVs as drug delivery vectors for potential treatment strategies.

## 1. Introduction

Amyotrophic Lateral Sclerosis (ALS) is a progressive neurodegenerative disease characterised by progressive motor neuron dysfunction leading to limb weakness, dysarthria, dysphagia and respiratory failure. The average survival is 2–4 years from symptom onset [1]. The incidence of ALS is rising and in recent studies is 1.6–3.8 per 100,000 [2]. Most cases (90%) of ALS are sporadic (sALS), with 10% of patients having a family history of ALS (fALS) [3]. There are over 50 genes known to cause or increase the risk of the disease, and a genetic cause has been reported in as high as 26.9% of patients in ALS registries [4]. A greater understanding of genetic risk factors has helped identify the multiple cellular processes affected in ALS which contribute to disease pathology. These include abnormalities in RNA processing, glutamate excitotoxicity, endoplasmic reticulum stress, mitochondrial dysfunction, protein homeostasis and endosomal transport/ extracellular vesicle secretion [5,6,7]. This review discusses the increasing knowledge of abnormalities in the regulation of extracellular vesicles in ALS, their effect on surrounding cells and how they are being developed as potential pathological markers and future therapeutic tools.

## 2. Extracellular Vesicles (EVs)

**Figure 1:**
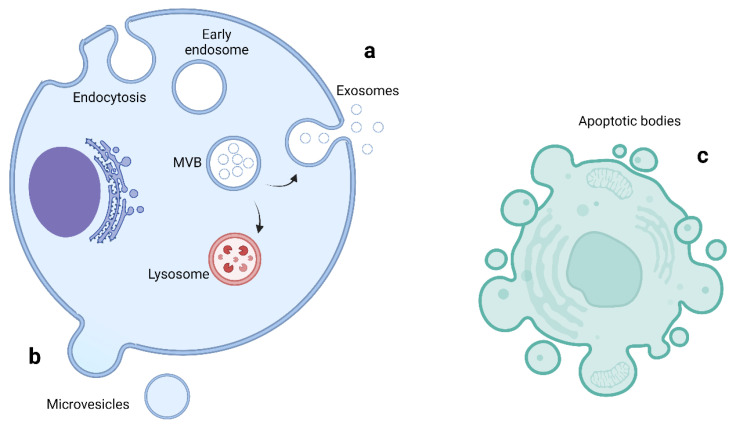
Biogenesis of extracellular vesicles. (**a**) Exosomes are formed by invagination of the endosomal membrane to form early endosomes. A second inward budding process leads to the formation of multivesicular bodies (MVBs). These are sorted to fuse either with lysosomes, leading to degradation, or with the plasma membrane, releasing their contents as exosomes. (**b**) Microvesicles are formed by the direct outward budding of the plasma membrane. (**c**) Apoptotic bodies are formed during the disintegration of the plasma membrane in dying cells. Figure created using biorender.com.

EVs are small vesicles enclosed in a lipid bilayer secreted from almost all cells and are detectible in a variety of biofluids [8,9]. They are involved in intercellular communication to both neighboring and distant cells through the transfer of lipids, proteins and genetic material [10,11]. EVs are stable in the circulation and have attracted much attention as potential non-invasive biomarkers in many pathological processes such as cancer, neurodegenerative diseases and cardiovascular disease [12,13,14,15,16]. They are classified into three main subgroups based on their size and biogenesis: exosomes, microvesicles and apoptotic bodies [9,17,18]. The biogenesis of extracellular vesicles is shown in Figure 1 and a comparison of EVs subtypes is summarized in Table 1.

### 2.1. Exosomes

Exosomes are the smallest of type of EV, typically 30–150 nm in diameter and derived from the endolysosomal pathway [9,12,19]. Endosome biogenesis begins with the inward budding of the endosomal membrane to form early endosomes. A second inward budding leads to the formation of late endosomes that contain intraluminal vesicles (IVL) [17,18]. The ILV membrane becomes enriched in tetraspanin proteins such as CD9 and CD63, which cluster into specialized units called tetraspanin enriched microdomains (TEMs) [8,20]. The tetraspanin proteins are involved in multiple processes such as cell adhesion, motility, membrane fusion, protein trafficking and signaling [20]. Late endosomes are referred to as Multivesicular bodies (MVB) [19]. The content of MVBs can be either degraded through fusion with lysosomes or released as exosomes through fusion with the plasma membrane [21]. The formation of exosomes from MVBs is mediated through two main pathways, the Endosomal Sorting Complex Required for Transport (ESCRT) dependent and ESCRT-independent systems [17]. The ESCRT dependent system consists of five core ESCRT complexes: ESCRT-0, -I, -II, -III and Vacuolar Protein Sorting 4–Vesicle Trafficking 1 (VPS4–VTA1), as well as accessory proteins such Tumor Susceptibility Gene 101 (TSG101) and ALG-2-interacting protein X (ALIX) [13,19,21]. These cytoplasmic multi-subunit complexes act together to sort ubiquitinated cargo into ILV and subsequent exosome formation and extracellular release [18,21].

The ESCRT independent pathway is a lipid dependent process [22]. Plasma membranes have an asymmetric lipid distribution with sphingomyelin and phosphatidylcholine enriched in the luminal side [23]. The hydrolysis of sphingomyelin to ceramide by sphingomyelinases (SMases) results in increased membrane fluidity and the cone shaped structure of ceramide results in negative curvature of the membrane and subsequent ILV formation [23,24]. A ceramide metabolite, sphingosine 1-phosphate (S1P) then activates receptors on MVBs to segregate ILVs for secretion as exosomes [25]. Support for this method of exosome formation is that inhibition of SMase reduces exosome secretion [26].

The cargo of exosomes consists of a variety of proteins, RNAs and lipids with their contents being determined by both ESCRT dependent and independent mechanisms [27]. Exosomes also allow for transfer of the plasma membrane and cytoplasmic components. Exosomal proteins include membrane transport and fusion proteins, heat shock proteins, tetraspanins, ESCRT proteins and cytoskeletal proteins [14]. They also contain messenger RNA (mRNA) and non-coding RNAs being particularly enriched with micro RNA (miRNA) [27]. Exosomes also contain a variety of lipids and are enriched in cholesterol, sphingolipids-particularly sphingomyelin and ceramide, and phosphatidylserine [28]. For all EVs there is heterogeneity in their contents based on the parent cell type and in addition EV cargo and secretion pattern changes in response to cellular stresses, e.g., oxidative stress, hypoxia or nutrient deprivation and cellular senescence [29,30,31].

### 2.2. Microvesicles

Microvesicles (MVs), also called microparticles or ectosomes, are larger EVs, typically 100–1000 nm in size [9,32]. They are formed directly from outward budding of the plasma membrane [9,19]. The mechanisms involved in MV formation are not as well understood as exosome biogenesis, but the outward budding is accompanied by changes in plasma membrane protein and phospholipid distribution particularly the flipping of phosphatidylserine to the outer membrane leaflet, which modulate changes in membrane curvature and rigidity [8,33]. Cellular structures involved in exosome biogenesis are also activated to create MVs such as ESCRT complexes and TSG101 [19]. A GTP-binding protein ADP ribosylation factor 6 (ARF6) activates phospholipase D resulting in the phosphorylation and activation of the myosin light chain resulting in the release of MVs into the extracellular space [8].

Similar to exosomes, MVs contain a variety of proteins, RNA and lipids. ARF6 is a key mediator for sorting cargo into MVs [30]. Typical proteins sorted into MVs are MHC class 1 proteins, vesicular SNARE proteins, ECSRT complex, heat shock proteins, mitochondrial proteins and ribosomal subunits [34,35]. MVs also contain RNA, with distinct RNA profiles compared to those present in exosomes and apoptotic bodies [36].

### 2.3. Apoptotic Bodies

Apoptotic bodies are larger EVs, usually between 500 and 5000 nm, with a small proportion between 50 and 500 nm and thus overlapping in size with the other EVs [8,9,37]. Apoptosis is a major mechanism of cell death [8]. It can be triggered by a wide range of physiological and pathological stimuli [37]. Apoptosis begins with condensation of nuclear chromatin, then membrane budding, followed by the disintegration of cellular content into membrane bound vesicles termed apoptotic bodies [8,37,38]. Apoptotic bodies consist of cytoplasm with tightly packed organelles, nuclear fragments, proteins, lipids and nucleic acid [39]. There are multiple plasma membrane changes during apoptosis including the oxidation of surface molecules to create binding sites for Thrombospondin and C3b, as well as the externalisation of phosphatidylserine to enable the binding of Annexin V. These changes facilitate phagocytosis by macrophages [8,38]. Like other EVs, apoptotic bodies circulate in the blood, are taken up by other cells, and are therefore involved in intercellular communication and the transfer of bioactive molecules [39].

Given the significant overlap in EV size, the lack of consensus on specific markers for each EV subtype, and the difficulty in determining the biogenesis pathway of EVs, the International Society of Extracellular Vesicles endorses the use of EV as the generic term for membrane bound particles released from cells and therefore this review will refer to all of the subtypes collectively as EVs [40].

### 2.4. Uptake of EVs

Once EVs are released from their cells of origin they accumulate in extracellular spaces, where they can remain intact for long periods as their membranes are resistant to breakdown [34]. EVs can then be taken up by recipient cells via several mechanisms, most commonly by endocytosis. Such endocytosis of EVs can occur by clathrin-mediated endocytosis, phagocytosis, micropinocytosis and lipid raft mediated endocytosis [41]. The EV membrane can also fuse directly with the plasma membrane and thus release its contents intracellularly [42]. A current area of research focus is to determine whether the uptake of EVs is targeted towards specific cells. Some studies have shown that EVs are taken up by any cell to which they are exposed [41,43]. However, EV uptake can also be a highly specific process requiring the expression of specific surface markers and ligands [44,45,46].

## 3. EVs in ALS

EVs are secreted from almost all cells and circulate freely around the body. They are able to cross the blood–brain barrier and therefore are a means of intercellular signalling to and from the central nervous system (CNS) [47]. EVs contribute to many physiological processes in the CNS, including: neural growth and development; CNS inflammation; the neuroprotective response to oxidative stress; and maintaining brain vascular integrity and post synaptic retrograde signalling [48,49,50,51,52]. EVs are also implicated in the pathological processes of neurodegeneration, and large numbers of studies have in recent years evaluated the role of EVs in neurodegenerative diseases, including ALS, Parkinson’s Disease (PD), Alzheimer’s Disease (AD), Huntington’s Disease and prion diseases [53].

### 3.1. ALS Associated Genes Involved in Vesicular Pathways

There are now over 50 potentially causal or disease modifying genes identified for ALS [5]. The most commonly identified gene mutation in European and North American patients is the hexanucleotide repeat expansion in *C9orf72*, accounting for up to a third of all identified pathogenic mutations [4]. Other common causal mutations are in the genes *SOD1*, *TARDBP*, *NEK1*, *FIG4* and *TBK1* [2,4,54]. There is wide geographical variation in the genetic causes of ALS. Familial ALS is much less frequent in China (1.2–2.7% of total cases), where mutations in *SOD1* are the most frequent genetic cause (25.3% of fALS), followed by *TARDBP* and *FUS* [55]. This is similar to Japan, where *SOD1* mutations are the most common cause of fALS (29.8%), followed by *FUS* and *TARDBP*, whereas *C9orf72* expansions are rare, identified in only 1.3% of fALS cases [56]. In Brazil, the most frequent genetic cause is *VAPB* (30% of all fALS) followed by *C9orf72* (22%) [57].

**Figure 2:**
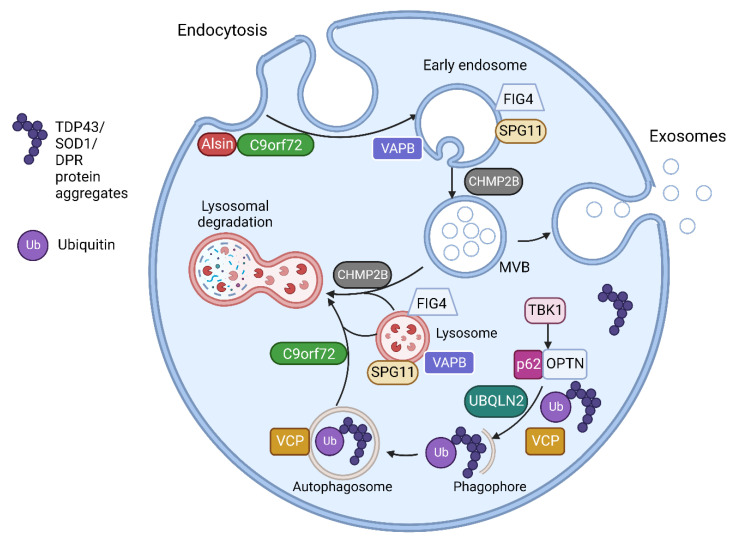
The endosomal and vesicular pathway involvement of proteins encoded by ALS related genes. Alsin activates Rab5 to promote endosomal fusion and subsequent endosomal trafficking. C9orf72 forms a complex with SMCR8 (Smith-Magenis Syndrome Chromosome Region, Candidate 8) and WDR41 (WD Repeat domain 41) proteins. This complex interacts with Rab GTPases including Rab5 in endosomal formation and trafficking. The C9orf72 complex also regulates various steps in autophagy including MVB-autophagosome and autophagosome-lysosome fusion as well as regulating several aspects of lysosomal biogenesis, pH and reformation. VAPB encodes Vesicle-associated membrane protein-associated protein B/C found in the endoplasmic reticulum (ER). This anchors complexes involved in lipid transfer from the ER to golgi and also the recycling of phosphatidylinositol-4-phosphate (PtdIns4P). VAPB mutations disrupt ER-golgi tethering and leads to PtdIns4P accumulation with subsequent accumulation of endosomes and dysfunctional lysosomes. FIG4 is required for the homeostasis of a signalling lipid phosphatidylinositol 3,5-bisphosphate (PI(3,5)P2), which is required for endosomal and lysosomal maturation. FIG4 mutations result in enlarged endosomes and lysosomes with impaired lysosomal function. CHMP2B is responsible for the formation of intraluminal vesicles within the MVBs and may participate in the proper fusion of MVB with the lysosomes and the autophagosomes. CHMP2B^intron5^ mutation results in accumulation of large endosomes and autophagosomes. VCP is involved in the initiation of autophagy and autophagosome maturation. TBK1 phosphorylates both optineurin (OPTN) and Sequestosome-1/p62, increasing their ability to bind to ubiquitinated cargo, initiating autophagy and delivery to autophagosomes. UBQLN2 interacts with LC3, a marker for starvation induced autophagy, to deliver ubiquitinated cargo to autophagosomes and is also recruited to OPTN containing vesicles. Spatacsin interacts with Rab5 for endosomal trafficking and maturation and contributes to lipid clearance from late endosomes and lysosomes. SPG11 mutations result in loss of spatacsin function, which leads to accumulation of lipids in lysosomes. Figure created using Biorender.com.

The multiple genes involved in ALS have a range of effects on multiple cellular processes including RNA processing, protein homeostasis, cytoskeletal dynamics, mitochondrial function, endosomal trafficking, autophagy and, important for this review, the formation of EVs [5,7]. Multiple ALS associated genes are involved in vesicular trafficking and EV regulation as shown in Table 2. The CHMP2B protein is an essential component of the ESCRT III complex, which is part of the machinery for MVB and EV formation, as discussed above [58]. *C9orf72*, *VAPB*, *FIG4*, *SPG11* and *ALS2* encode proteins which are involved in intracellular trafficking of vesicles [59,60,61,62,63,64]. Optineurin and SQSTM1/p62 proteins are both autophagy receptors and are activated through phosphorylation by TBK1 [65]. Autophagy and EV function are closely linked, with the level of autophagy in cells closely related to the secretion and transport of EVs [66,67]. Table 2 lists some of the proposed pathogenic mechanisms for mutations of genes having functions related to vesicular pathways (also illustrated in Figure 2).

### 3.2. EV Mediated Transfer of Misfolded Proteins and miRNAs in ALS

**Figure 3:**
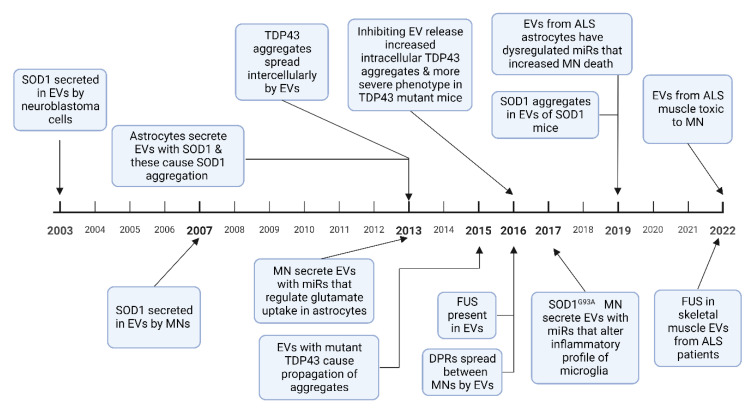
A timeline of experimental evidence of how EVs are involved in the spread of misfolded proteins, and how miRNA alter cellular phenotypes, in ALS cell-based and animal models. Abbreviations: MN-motor neuron, DPR-dipeptide repeat proteins [82,83,84,85,86,87,88,89,90,91,92,93,94]. Figure created using Biorender.com.

A pathological hallmark of ALS is TAR DNA-binding protein 43 (TDP 43) positive inclusions, which have been identified in brain stem and spinal cord tissue in over 97% of patients at postmortem [75]. SOD1, fused in sarcoma (FUS), and dipeptide repeat proteins (DPRs) from the *C9orf72* intronic hexanucleotide repeat expansion, also aggregate to form protein inclusions [76]. These misfolded proteins have been demonstrated to spread between cells in a prion-like manner and induce further protein misfolding [77]. This mechanism has been used to explain the contiguous spread of disease that is often seen in ALS, where the disease spreads to adjacent neuroanatomical segments [78]. EVs have been shown to contain aberrant protein aggregates in cell and animal models of ALS as well as in patients with ALS. Evidence is growing to support the hypothesis that EVs can spread pathological misfolded proteins between cells in ALS and that these EVs can exert deleterious effects on recipient cells, discussed in Section 4 below. EVs have also been shown to mediate the transfer of RNAs, particularly miRNA, which can alter gene expression in recipient cells, potentially also of relevance to the progressive spread of neurodegeneration in ALS. Studies in other neurodegenerative diseases have also found that pathological proteins such as prion protein, amyloid-β, α-synuclein, and tau propagate via exosomes [79,80,81]. A timeline of discoveries in cell based and animal models of these roles of EVs in ALS is shown in Figure 3.

#### 3.2.1. SOD1

The first evidence that EVs could spread misfolded proteins was in 2003 when SOD1 was shown to be excreted by EVs in SK-N-BE neuroblastoma cells [82]. Astrocytes with mutant SOD1 overexpression showed increased EV secretion compared to wild type cells; these secreted EVs were taken up by motor neurons, inducing cell death [84]. Misfolded SOD1 aggregates spread between NSC-34 motor neuron-like cells via EVs, causing cell rupture and cell death [83,95]. Once the SOD1 aggregates are introduced to neural cells, they result in a self-perpetuating induction of further SOD1 aggregation and transfer between cells [96]. These results have also been replicated in animal models, with CNS derived EVs in SOD1^G93A^ mice containing misfolded SOD1 aggregates [92]. However, while mutant SOD1 aggregates are secreted by EVs, other studies have reported impaired secretion of mutant SOD1 compared to the wild type protein in NSC-34 motor neuron-like cells and rat microglial cells, with a proposed underlying mechanism of dysfunctional secretory pathways as a result of golgi fragmentation and ER stress [97,98].

#### 3.2.2. TDP 43

Multiple studies demonstrate that TDP 43 is transferred intercellularly via EVs. Insoluble TDP 43 aggregates from ALS or FTD brain tissue resulted in intracellular accumulation of TDP 43 and cell death when added to SH-SY5Y neuroblastoma cells [85]. The same study also showed that TDP 43 aggregates can transfer between cells via EVs. CSF EVs in patients with ALS and ALS/FTD contain TDP 43 and these EVs have been shown to cause propagation of TDP 43 aggregates when added to U251 glioblastoma cells [87]. EVs isolated from human ALS brain tissue also caused TDP 43 accumulation and propagation in Neuro2a mouse glioblastoma cells [88]. In the same study, EV release was inhibited by a sphingomyelinase inhibitor, which resulted in increased TDP 43 aggregates in the Neuro2a cells and also exacerbated the clinical phenotype of transgenic mice expressing human mutant TDP-43^A315T^. This suggests that while EVs play a key role in the propagation of TDP 43 proteinopathy, the inhibition of EV secretion may precipitate greater intracellular accumulation of pathological aggregates [88]. Free TDP 43 can also be taken up by cells, but it has been shown in human embryonic kidney 293 (HEK-293) cells that EVs containing TDP 43 are preferentially taken up compared to free TDP 43, and thus have potential to spread and exert greater cellular toxicity [99].

#### 3.2.3. FUS

There are few studies analysing mutant FUS or FUS binding partners. Analysis of HEK cells expressing mutant FUS^R521G^ confirmed that FUS is present in EVs and that many FUS binding partners are components of EVs [89]. FUS and several FUS binding partners were also observed in EVs derived from skeletal muscle samples from sALS patients without FUS mutations [94].

#### 3.2.4. Dipeptide Repeat Proteins

DPRs are a group of 5 protein complexes formed as a result of repeat-associated non-AUG (RAN) translation of the *C9orf72* intronic hexanucleotide repeat expansion [100]. Such DPRs have been shown to spread intercellularly via EVs in spinal motor neurons derived from induced pluripotent stem cells (iPSCs) expressing the expanded hexanucleotide repeat [90].

#### 3.2.5. RNA Transport by EVs

While multiple types of RNA have been found in EVs, most is known about miRNAs. These are short non-coding RNAs which have gained great attention due to their ability to modify gene expression in recipient cells [101]. MiRNAs are stable in the circulation and are found in a range of body fluids including serum, plasma, urine and CSF [102]. There is also evidence of their transport in EVs between cells [103]. EVs contain miRNA profiles that are vastly distinct from their host cell from which they originate [104]. Multiple miRNAs have been identified as dysregulated in vitro in ALS models involving several different cell types. EVs have been shown to transmit miR-124-3p from neurons to astrocytes, which regulates the glutamate uptake of astrocytes [105]. A study on astrocytes derived from iPSCs from C9orf72 ALS patients compared with iPSCs from healthy controls found that the EV miRNA content was dysregulated in ALS, with 64 dysregulated miRNAs, and downregulation of miR-494-3p as the most significant change [93].

There are fewer in vitro studies of messenger RNA (mRNA) expression in EVs in ALS. Similar to miRNA, mRNA expression in EVs from iPSC derived motor neurons is markedly different from that of the iPSC cells themselves, being enriched for genes regulating cellular metabolism and protein homeostasis [106].

## 4. Effects of ALS EVs on Recipient Cells

EVs derived from multiple cell lines have been demonstrated to contribute to neurotoxicity in cell models of ALS. Skeletal muscle EVs from patients with ALS are toxic to motor neurons in culture, causing shortened, less branched neurons with disrupted localisation of RNA and RNA processing proteins, and increased cell death [94]. Blockade of EV uptake by first incubating the EVs with anti-CD63 antibody resulted in increased motor neuron survival. Motor neurons treated with EVs from astrocytes overexpressing mutant SOD1 had reduced survival, proportionate to the concentration of EVs applied [84]. EVs extracted from CSF of patients with ALS and ALS/FTD resulted in increased expression of TDP43 C-terminal fragments and induced apoptosis and autophagy when applied to U251 glioblastoma cells [87].

EVs have also been shown to alter astrocyte, microglial and monocyte function in ALS. A study of co-cultured neurons and astrocytes reported that transfer of miR 124-a from neurons to astrocytes through EVs resulted in increased GLT1 expression in astrocytes, with resultant increase in glutamate uptake, preventing excitotoxicity [86]. The same group subsequently performed stereotactic injection of miR 124-a into the spinal cord ventral grey matter of SOD1^G93A^ mice and this resulted in a 30–40% increase in GLT1 levels, demonstrating a potential method for limiting glutamate mediated excitotoxicity [86]. NSC-34 cells transfected with SOD1^G93A^ produce EVs containing increased miR 124 expression and in co-culture with N9 microglial cells resulted in NF-κB activation, upregulation of several inflammatory cytokines and a 50% reduction in microglial phagocytosis [91]. A subsequent study reported that microglial cells overexpressing SOD1^G93A^ secreted EVs with upregulated miR 155 and 146a, miRNAs which are both involved in the regulation of the NF-κB inflammatory pathway [107]. Another report on astrocytes derived from iPSCs from mutant C9orf72 ALS patients found that EVs from the mutant C9orf72 astrocytes were toxic to motor neurons and identified miRNA dysregulation as discussed in Section 3.2.5 [93]. Subsequent treatment of the cells with miR-494-3p resulted in a 25% increase in motor neuron survival. Taken together, these studies provide strong data that miRNAs delivered by EVs induce changes in recipient cells, that may contribute to disease pathology in ALS.

EVs containing mutant TDP-43 have also been shown to induce changes in peripheral monocytes, with increased secretion of inflammatory cytokines such as IL-6, IL-10 and IL-1 but this secretion was impaired in ALS monocytes compared to healthy controls [108].

### 4.1. EVs as Biomarkers in Patients with ALS

The results of the above studies have led to much interest in the possibility of using EVs as biomarkers in ALS. EVs have many beneficial characteristics for this. They penetrate the blood–brain barrier in a bidirectional manner, providing a means of communication to and from the CNS [109]. They are stable in the peripheral circulation and protect their cargoes from degradation [110]. Isolation of EVs based on their surface markers allows identification of their specific cellular origin, including neural cell derived EVs [53]. The studies evaluating EVs as biomarkers in ALS are summarised below.

Studies in ALS patients have shown that TDP43, SOD1 and FUS levels are elevated in EVs isolated from plasma and cerebrospinal fluid (CSF), listed in Table 3. Motor cortex EVs from human ALS post-mortem tissue showed upregulation of several RNA binding proteins [111]. Inflammatory cytokines have also been identified, with increased levels of interleukin 6 found in astrocyte derived EVs in patients with ALS [112]. Recent studies have aimed to analyse the whole proteome of EVs in ALS. Table 3 also lists the results of two such proteomic studies performed on CSF derived EVs of patients with ALS. Interleukin 6 and SOD1 protein levels were also shown to correlate with the rate of change of ALSFRS-R in patients, demonstrating that EV biomarkers may be useful to measure disease progression.

The results of eight studies analysing the miRNA content of EVs in patients with ALS are also summarised in Table 3. Five of these analysed the total miRNA content of EVs, two evaluated specific miRNAs, miR-27a and miR-124, chosen based on previous in vitro studies, and another validated a panel of 8 miRNAs from a previous study on patients with ALS. Seven studies reported altered miRNA expression in ALS. Overall there was very limited overlap in the results between studies, with only the upregulation of miR-3919-3p and downregulation of both miR-4454 and miR-4286 being shown in more than one study [113,114,115,116]. There were also multiple conflicting results between studies, with let-7c-5p, miR 9-5p, miR 199a-3p, miR 199a-5p and MiR 4508 having been reported to be both upregulated and downregulated in ALS [113,114,115,116]. Banack et al. reported 8 dysregulated miRNA in their 2020 study [116]. They subsequently performed a further validation study in a separate cohort of 50 patients with ALS and 50 controls, and confirmed their previous findings of upregulated miR 4a-5p, miR 146a-5p and downregulated miR 4454, miR 10b-5p and miR 29b-3p, and have suggested these may be useful as disease biomarkers [117].

Studies on miRNA from serum, plasma and CSF from patients with ALS compared to healthy controls have also reported a wide range of miRNA as potentially relevant in ALS [118,119,120,121]. As above, there is limited overlap of significant findings between studies and often conflicting results. One of the more consistent findings is upregulated miR 206, which is involved in muscle repair, regeneration and the promotion of new neuromuscular junctions following denervation [122]. However, this is not specific to ALS and is seen in other conditions such as spinal bulbar muscular atrophy (SBMA), spinal muscular atrophy (SMA) and in muscular dystrophies [123,124,125]. Promising miRNAs reported in multiple studies affect genes regulating neurodegeneration and apoptosis (miR-338, miR-142, miR-183 and let-7d) or muscle (miR-206, miR-133a, miR-133b and miR-27a) [120]. Sproviero et al. found that MiR 133a and miR 206 were among 45 miRs increased ALS compared to controls in both small and large plasma EVs [114]. The only other overlap in miRs from EV studies with other biofluds are altered expressions of miR 338, this being downregulated in EVs, but reported to be upregulated in plasma [115,126].

There have been two studies analysing the mRNA profile of EVs in patients with ALS. Otake et al. found 133 upregulated and 410 downregulated genes in EVs extracted from CSF of 4 ALS patients. Gene ontology analysis showed enrichment in mRNAs involved in the ubiquitin-proteasome pathway, oxidative stress response and unfolded protein response [127]. Sproviero et al. reported 542 upregulated genes and 88 downregulated genes in plasma EVs from ALS patients. Gene ontology analysis from these results showed enrichment of genes involved in the regulation of transmembrane transport, leukocyte chemotaxis and transcription regulation [128].

Lipid dysregulation is gaining attention as a key feature in ALS, with multiple studies showing alterations in many lipid classes, particularly sphingomyelin and ceramide, in ALS [129,130,131,132]. As with other substrates, the lipid composition of EVs is different from host cells. There is enrichment by a factor of 2–3 for cholesterol, sphingomyelin, glycosphingolipids and phosphatidylserine in EVs analysed from multiple cell lines [133]. The high content of cholesterol and sphingomyelin allows for tighter lipid packaging in membranes giving greater structural rigidity to EVs and resistance to physicochemical changes. Additionally, cholesterol interacts with sphingomyelin to form lipid rafts, which are lipid and protein rich domains highly involved in signalling pathways [134]. Bioactive lipids such as sphingolipids and eicosanoids can also be transferred between cells by EVs [135]. Few lipidomic studies have been performed in EVs and none on EVs from patients with ALS [136]. There has only been one study analysing the lipid content of EVs in patients with ALS. Morasso et al. used Raman spectroscopy, a form of vibrational spectroscopy, and found the total lipid content of EVs was increased in patients with ALS, however there was no information on specific lipids [137]. There is very limited information on the lipid content in EVs in ALS and this area merits further investigation.

**Table 3:** Studies showing the use of EVs as biomarkers in patients with ALS. Abbreviations: AD—Alzheimer’s Disease, DC—disease control, FTD—Frontotemporal dementia, HC—Healthy control, SBMA—spinal bulbar muscular atrophy, MD—muscular dystrophy, PD—Parkinson’s Disease, LMVs—Leukocyte derived microvesicles, ALSFRS—ALS functional rating scale, IL-6—Interleukin-6, IP—immunoprecipitation, UC—ultracentrifugation, NBI—nickel based isolation, PEG—polyethylene glycol, NOC2l—Nucleolar complex protein 2 homolog, PDCD6IP—Programmed cell death 6-interacting protein, VCAN—Versican core protein, BLMH—bleomycin hydrolase, HSP90—Heat shock protein 90, PPIA—peptidyl-prolyl cis-trans isomerase A.

Study	Source	Isolation Method	Patients	Metabolites Analysed	Results
Protein					
Feneberg et al. 2014 [138]	CSF-Exosomes	UC	9 ALS, 4 FTD, 8 HC	TDP 43	TDP 43 detectable in EVs but not different between groups
Sproviero et al. 2018 [139]	Plasma- exosomes and MVs	Filtration and UC	30 ALS, 30 HC	SOD1, TDP 43 and FUS	Increased SOD1, TDP 43, phosphorylated TDP 43 and FUS in ALS MVs
Chen et al. 2019 [112]	Plasma- astrocyte derived exosomes	Polymer based precipitation followed by IP with anti-ACSA-1	40 ALS, 39 HC	IL-6	IL-6 increased in ALS and correlated with rate of ALSFRS change
Sproviero et al. 2019 [140]	Plasma-Leukocyte, endothelial, platelet and erythrocyte derived MVs	UC followed by IP with anti-CD45	40 ALS, 36 HC, 28 AD	SOD1, TDP 43	Misfolded SOD1 detectable in plasma LMVs SOD1 levels in LMVs correlated with rate of ALSFRS change in slow progressors
Chen et al. 2020 [141]	Plasma-exosome	IP with anti CD63	18 ALS	FUS	FUS present and increased at 3 and 6 months
Hayashi et al. 2020 [142]	CSF-exosomes	Size exclusion chromatography	3 ALS, 3 NPH	334 proteins	NOC2l, PDCD6IP, VCAN increased in ALS 11 proteins decreased
Thompson et al. 2020 [143]	CSF- EVs	Ultrafiltration liquid chromatography	12 ALS, 5 HC	1020 proteins	Downregulation of BLMH
Pasetto et al. 2021 [144]	Plasma- EVs	UC and NBI	106 ALS, 36 HC, 32 SBMA, 28 MD	TDP 43, HSP90 and PPIA	HSP90 reduced in ALS compared to HC and SBMA ALS EVs smaller than SBMA
Micro RNA					
Xu et al. 2018 [145]	Serum-exosomes	membrane affinity spin columns	10 ALS, 20 HC	miR-27a-3p	miR-27a-3p downregulated in ALS
Katsu et al. 2019 [113]	Plasma- neural derived EVs	PEG precipitation followed by IP with anti CD171	5 ALS, 5 HC	332 miRNAs	13 upregulated miRNAs, greatest increase in 4736, 4700-5p, 1207-5p, 4739, 4505 17 downregulated miRNAs
Saucier et al. 2019 [115]	Serum-EVs	Vn96 peptide affinity capture	14 ALS, 12 HC	Total miRNA profile	Upregulated 532-3p, 144-3p, 15a-5p, 363-3p and 183-5p 22 downregulated miRs, greatest reduction in 4454, 9-1-5p and, 9-3-5p, 338-3p and 9-2-5p
Banack et al. 2020 [116]	Plasma-neural derived	PEG precipitation followed by IP with anti CD171	20 ALS, 20 HC	34 miRNAs	Upregulated 146a-5p, 199a-3p, 151-a-3p, 151a-5p, 199a-5p Downregulated 4454, 10b-5p, 29b-3p
Yelick et al. 2020 [146]	CSF-EVs	Polymer based precipitation	14 ALS, 9 DC, 9 HC	miR-124-3p	miR-124-3p correlated with ALSFRS in males with ALS
Pregnolato et al. 2021 [147]	Plasma- exosome miRNA	Polymer based precipitation	7 ALS, 3 HC	179 miRNAs	No difference in miRNA expression
Sproviero et al. 2021 [114]	Plasma—large and small EVs	Filtration and UC	6 ALS, 9 FTD, 6 AD, 9 PD, 6 HC	total miRNA	45 upregulated and 22 downregulated miRNA in both small and large EVs in ALS vs. HC
Banack et al. 2022 [117]	Plasma-neural derived	PEG precipitation followed by IP with anti CD171	50 ALS, 50 HC	8 miRNAs	Upregulated 4a-5p, 146a-5p Downregulated 4454, 10b-5p and 29b-3p
Messenger RNA					
Otake et al. 2019 [127]	CSF- EVs	membrane affinity spin columns	4 ALS, 4 HC	5006 mRNAs	133 upregulated 410 downregulated
Sproviero et al. 2022 [128]	Plasma—large and small EVs	Filtration and UC	6 ALS, 9 FTD, 6 AD, 9 PD, 6 HC	Total mRNA	542 upregulated 88 downregulated
Lipids					
Morasso et al. 2020 [137]	Plasma EVs	UC	20 ALS, 20 HC	Raman spectra	Total lipid content increased in ALS Phenylalanine decreased

### 4.2. Challenges in Developing EV Biomarkers

There are multiple difficulties in developing EVs as biomarkers and multiple possible reasons for the general lack of agreement that is often reported between studies. This includes the various biofluids investigated, which included CSF, plasma, and serum to isolate EVs. Additionally, the studies have used multiple EV extraction techniques and downstream purifications for example to isolate astrocyte or neural derived EVs. There are many techniques available for isolating EVs, each with distinct advantages and disadvantages as shown in Table 4. The commonly used techniques are ultracentrifugation, polymer based precipitation, size exclusion chromatography, ultrafiltration and immunoaffinity capture [148,149]. Given the multiple different isolation and purification techniques available, it will be crucial for upcoming studies to follow the International Society of Extracellular Vesicles guidelines on characterisation of EVs to maximise validity and enable a more reliable comparison between the obtained findings [40]. This will allow future validation studies to more robustly investigate proposed EV biomarkers in ALS.

## 5. Therapeutic Application of EVs in ALS

There has been great interest in the potential use of EVs as a non-invasive method to deliver therapeutics including proteins, genetic material and drugs, in neurodegenerative diseases [150]. The investigation of EVs as therapeutic vectors is growing with at least 20 phase 1/2 clinical trials registered in patients with cancer, SARS-CoV-2, and Alzheimer’s disease, with treatments including stem cell derived EVs, autologous EVs or drug loaded EVs [151]. There are several properties of EVs which suggest that they could be useful in therapeutics in neurodegenerative diseases such as their ability to cross the blood–brain barrier, their low tendency to evoke an immune response and the potential for manipulation of cell surface markers to limit off target effects [152,153]. However, there are also challenges in development of EVs for this use, such as batch-to-batch variation in their synthesis, the current lack of cost-effective large scale production protocols or of robust, reproducible methods for drug loading [151]. EVs have been used in murine models of PD and AD to deliver small interfering RNAs (siRNA) to reduce pathological protein accumulation. EVs with α-Synuclein siRNA were peripherally injected into α-Synuclein^S129D^ transgenic mice. This decreased the level of α-Synuclein aggregates in brain regions pathologically affected in PD [154]. EVs containing BACE1 siRNAs have also been used in C57BL/6 mice resulting in an over 60% reduction in BACE1 mRNA and a 55% decrease in β-amyloid 1-42 levels, a key component of plaques in AD pathology [155].

Studies in ALS have used EVs taken from mesenchymal stem cells (MSCs). The capacity of MSCs to replicate, differentiate, secrete neuroprotective factors and produce new cells to replace damaged cells, has led to multiple phase 1/2 clinical studies investigating their potential use in ALS [156]. However, challenges to the use of MSCs include dosing issues, variation in the differentiation state of the cells and the route of administration [157]. As it is now recognised that MSCs exert much of their action through secretion of EVs, investigation of the MSC secretome and EVs, as a cell-free therapeutic approach, is now being explored [158].

EVs from adipose derived stem cells (ADSCs) have been investigated in ALS cell models. Healthy human ADSCs were added to murine SOD1^G93A^ NSC cells resulting in slower SOD1 aggregation and improved mitochondrial function [159]. EVs from Murine ADSCs have also been added to NSC-34 cells overexpressing SOD1^G93A^, SOD1^G37R^ and SOD1^A4V^, which were challenged to oxidative damage with hydrogen peroxide. Treatment with EVs reduced oxidative damage, increased cell viability and improved mitochondrial function [160,161]. A subsequent study by the same group confirmed their previous findings and reported proteomic analysis on the ADSC EVs, identifying 189 proteins. Gene ontology analysis showed the most significant biological processes of the identified proteins to be cell adhesion, negative regulation of apoptosis and positive regulation of cell proliferation [162]. The group also tested intravenous and intranasal administration of ADSC EVs to SOD1^G93A^ mice. The EVs were labelled with ultra-small superparamagnetic iron oxide nanoparticles and MRI confirmed the EVs did penetrate the blood–brain barrier and were deposited in the brain. The mice showed improved motor performance compared to placebo and had greater preservation of lumbar motor neurons, neuromuscular junctions and muscle fibres [163].

EVs isolated from human bone marrow-derived endothelial progenitor cells have also been shown to reduce damage in a murine brain endothelial cell ALS model [164]. Giunti et al. modified EVs from bone marrow derived MSCs by first treating the MSCs with interferon-γ. Treating the MSCs with interferon-γ resulted in upregulation of multiple microRNAs in EVs including miR-466q and miR-467f, which can reduce microglial activation through inhibition of the p38 mitogen-activated protein kinase pathway. The EVs reduced the levels of mRNA for Tumour Necrosis Factor and Interleukin 1b in SOD1^G93A^ microglial cells [165]. This study showed that EV content can in principle be altered to exert the desired effect on target cells.

## 6. Conclusions

EVs have multiple potential applications in the investigation of pathology, early (perhaps pre-clinical) diagnosis, and therapeutic management of patients with ALS. They have been shown to play a role in disease pathogenesis through the transfer and subsequent intracellular accumulation of pathological proteins such as TDP-43, SOD1 and FUS. Multiple studies have reported dysregulation of the protein and microRNA cargo of EVs in cell and animal models of ALS and in patients. Some studies have shown correlation of EV content with markers of disease progression and there is the exciting scope, with further validation studies, to develop EVs as diagnostic and prognostic biomarkers. However, the wide variety of EV sources and isolation methods have limited the reproducibility and comparability of studies to date. EVs also hold tantalizing promise as therapeutic agents in ALS and other neurodegenerative diseases through the delivery of neuroprotective factors in EVs derived from MSCs or as drug delivery vectors.

## Figures and Tables

**Table 1:** Comparison of Extracellular Vesicles subtypes.

	Exosomes	Microvesicles	Apoptotic Bodies
Size (nm)	30–150	100–1000	50–5000
Biogenesis	Derived from inward invagination of endosomes to forms MVB, released once MVB fuses with plasma membrane	Outward budding of plasma membrane	Membrane budding and disintegration during apoptosis
Contents	membrane transport and fusion proteins, heat shock proteins, tetraspanins, ESCRT proteins and cytoskeletal proteins RNA-particularly mRNA and miRNA, lipids including cholesterol, sphingomyelin and phosphatidylserine	Overlap with exosomes and MHC class 1 proteins, vesicular SNARE proteins, mitochondrial proteins and ribosomal subunits. RNA profile distinct from exosomes, lipids including cholesterol, sphingomyelin and phosphatidylserine	cytoplasm with tightly packed organelles, nuclear fragments, proteins, lipids and nucleic acid
Markers	Tetraspanins (CD63, CD9, CD81), HSP70, ALIX, TSG101, flotillin 1	ARF6, Integrins, selectins, CD40	Annexin V, Thrombospondin, C3b

**Table 2:** ALS genes with roles in vesicular transport and EV regulation. Abbreviations: DPR—dipeptide repeat proteins, RBP—RNA binding proteins, UPS—ubiquitin proteasome system, NF-κB—nuclear factor kappa B.

Gene	Proteins	Molecular Pathways Affected
*C9orf72* [59,68]	C9orf72 short and long isoforms	Loss of function in vesicle trafficking, autophagy and endo-lysosomal pathway Gain of toxicity with development of RNA foci and DPR
*VAPB* [62,63]	Vesicle-associated membrane protein-associated protein B/C	Aggregation of VAPB protein, altered autophagy and vesicular transport, accumulation of RBPs
*FIG4* [60]	Polyphosphoinositide phosphatase	Loss of function in trafficking of endosomal vesicles to golgi and autophagy regulation
*ALS2* [61]	Alsin	Alteration of Rab5-mediated pathway with dysregulation of endosomal trafficking Altered trafficking of AMPA receptors causing glutamate toxicity
*CHMP2B* [58]	Charged multivesicular body protein 2b	Dysfunction of autophagy and endo-lysosomal pathway, resulting in accumulation of enlarged endosomes and autophagic organelles
*SPG11* [64]	Spatacsin	Impaired autophagy, lipid sorting in late endosomes and lysosomal dysfunction with lipid accumulation
*SQSTM1* [69]	Sequestosome-1/p62	Dysfunction of autophagy and protein degradation through UPS
*OPTN* [70]	Optineurin	Golgi fragmentation, impaired autophagy and vesicular transport Loss of inhibitory action on NF-κB leading to abnormal inflammatory response
*UBQLN2* [71]	Ubiquilin 2	Impaired protein degradation via UPS and dysfunction of autophagy and endo-lysosomal pathway
*VCP* [72,73]	Valosin Containing Protein	Impaired protein degradation via UPS and dysfunction of autophagy and endo-lysosomal pathway
*TBK1* [74]	Tank Binding Kinase 1	Dysregulation of multiple autophagy pathways

**Table 4:** Comparison of the advantages and disadvantages of commonly used EV isolation techniques [148,149].

	Ultracentrifugation	Polymer Based Precipitation	Size Exclusion Chromatography	Ultrafiltration	Immunoaffinity
Advantages	High purity Can collect different size EVs	No specialised equipment Quick High yield High throughput	High purity Low cost Quick	Low cost Quick	Can be used to separate EVs of different origins High purity
Disadvantages	Low yield Specialised equipment Requires large sample Low throughput	Low purity	Low yield	EV clogging and trapping Low yield Low purity	Costs EV markers require optimisation Elution steps may damage EV structure

## Data Availability

Not applicable.
